# Distinct cerebral cortical microstructural changes in idiopathic normal-pressure hydrocephalus

**DOI:** 10.3389/fneur.2025.1618788

**Published:** 2025-08-13

**Authors:** Myong Hun Hahm, Shin Young Jeong, Suhyun Kim, Sang-Woo Lee, Ki-Su Park, Eunhee Park, Mi-Yeon Eun, Uicheul Yoon, Kyunghun Kang

**Affiliations:** ^1^Department of Radiology, Daekyung Imaging Center, Daegu, Republic of Korea; ^2^Department of Nuclear Medicine, School of Medicine, Kyungpook National University, Daegu, Republic of Korea; ^3^Department of Biomedical Engineering, Daegu Catholic University, Gyeongsan-si, Republic of Korea; ^4^Department of Neurosurgery, School of Medicine, Kyungpook National University, Daegu, Republic of Korea; ^5^Department of Rehabilitation Medicine, School of Medicine, Kyungpook National University, Daegu, Republic of Korea; ^6^Department of Neurology, School of Medicine, Kyungpook National University, Daegu, Republic of Korea

**Keywords:** idiopathic normal-pressure hydrocephalus, mean diffusivity, diffusion tensor imaging, magnetic resonance imaging, Alzheimer’s disease

## Abstract

**Objective:**

The aims of the study were to investigate differences in cortical mean diffusivity (MD) among idiopathic normal-pressure hydrocephalus (INPH) patients, Alzheimer’s disease (AD) patients, and healthy controls, and to analyze mean MD among INPH and AD groups in INPH-specific areas showing distinctive cortical MD changes for distinguishing INPH from AD.

**Methods:**

Forty-two INPH patients, 51 AD patients, and 23 healthy controls were imaged with MRI, including diffusion tensor imaging MR images, for surface-based analysis across the entire brain.

**Results:**

Compared with healthy controls, INPH patients showed a statistically significant reduction in MD in the high convexity of the frontal, parietal, and occipital cortical regions. We designate these clusters of lower MD as INPH MD LOW ROI. Additionally, a significant increase in MD, mainly in the ventromedial frontal cortex, ventrolateral frontal cortex, supramarginal gyrus, and temporal cortical regions, was observed in the INPH group relative to the control group. We designate these clusters of higher MD as INPH MD HIGH ROI. INPH patients showed significantly lower mean MD in INPH MD LOW ROI and higher mean MD in INPH MD HIGH ROI than AD. The mean MD of INPH MD LOW ROI had an AUC of 0.857 for differentiating INPH from AD.

**Conclusion:**

A distinctive pattern of cortical MD changes was found in INPH patients, and cortical regions of low MD distinguished INPH from AD with good diagnostic sensitivity and specificity. Our findings suggest microstructural changes in cortical integrity can help differentiate INPH and AD in elderly patients.

## Introduction

Idiopathic normal-pressure hydrocephalus (INPH) is considered a treatable neurologic condition associated with normal cerebrospinal fluid (CSF) pressure, ventricular dilatation, and symptoms of gait disturbance, cognitive impairment, and urinary dysfunction (1).

The cortex is usually overlooked and white matter is often the main focus of investigation when researching pathophysiological mechanisms in INPH (2). Nevertheless, a few studies have suggested that neuronal degeneration occurs both distally (Wallerian degeneration) and proximally (dying back) when axons in the brain are damaged (3). These mechanisms can cause cortical deterioration in regions connected to the damaged white matter (3). Further, in one study, a characteristic regional pattern of cortical perfusion changes was observed in the INPH patients relative to controls (4). Moreover, a perfusion deficit can be connected with structural deterioration (5). Consequently, we hypothesized that in INPH patients cortical degeneration may be as important as degeneration in white matter.

In the past 10 years, there has been increased interest in diffusion tensor imaging (DTI), due to its sensitivity to microstructural properties of brain parenchyma (6). Further, DTI is also used to study microstructural changes in gray matter (6). Moreover, mean diffusivity (MD) is often investigated in studies of gray matter as the cortex is primarily an isotropic structure (6). The promise of investigating microstructural changes in gray matter using DTI has been shown in Alzheimer’s disease (AD) (6, 7). These reports demonstrated that MD in gray matter is generally higher in AD patients when compared to healthy controls, and that MD may become a promising imaging biomarker (6, 7). Furthermore, AD is the most frequent cause of dementia in the elderly, and while ventriculomegaly is the central characteristic of INPH, it is also observed in AD (8). AD patients exhibit diffuse cerebral atrophy that can result in secondary ventricular enlargement. INPH with non-obstructive ventricle enlargement can be challenging to distinguish from AD with ex vacuo ventricular enlargement when based on typical MRI findings alone (1). In addition, INPH diagnosis is made even more challenging by its clinical variability (1). That said, timely INPH diagnosis is crucial as INPH is regarded as a treatable neurodegenerative disease.

In this study, we utilized surface-based DTI analysis to investigate differences in cortical MD among INPH patients, AD patients, and healthy controls. We analyzed mean MD among INPH and AD groups in INPH-specific areas showing distinctive cortical MD changes for distinguishing INPH from AD.

## Methods

### Participants

INPH patients who visited the Center for Neurodegenerative Diseases of Kyungpook National University Chilgok Hospital, South Korea, from June 2017 to March 2021 were prospectively recruited. The INPH diagnosis was made using Relkin et al. (1) criteria. A lumbar tap removing 30–50 mL of CSF was done on each INPH patient. After the CSF tap, patients were re-evaluated with the Korean-Mini Mental State Examination (K-MMSE), the INPH Grading Scale (INPHGS), and the Timed Up and Go Test (TUG). Gait changes were evaluated multiple times over 7 days following the tap, and changes in cognition and urination were evaluated at 1 week. CSFTT response was defined using these 3 major scales (9). INPH patients who had a positive response to the CSFTT according to the Japanese guidelines for INPH were enrolled (9). AD patients and healthy controls were chosen randomly from our hospital and were matched to INPH patients according to age. AD was diagnosed using McKhann et al. (10) criteria. We included participants with clinically probable AD dementia (10). The criteria for healthy controls were as follows: normal neurological status on examination; no active neurological, systemic, or psychiatric disorders; and ability to function independently. Global cognition of healthy controls was also assessed using the K-MMSE.

### MRI imaging acquisition

The MRI data were obtained using a 3.0 T system (GE Discovery MR750, GE Healthcare). The DTI data were obtained using a single-shot echo-planar acquisition (45 non-collinear diffusion gradient directions, TR = 9,900 ms, TE = 76 ms, matrix = 128 × 128, field of view = 240 mm, slice thickness = 2.0 mm without a gap, flip angle = 90°, and b-factor = 600 s/mm^2^). Three-dimensional T1-weighted, sagittal, and inversion-recovery fast spoiled gradient echo (IR-FSPGR) MRI images of the whole head, designed to optimally discriminate between brain tissues (sagittal slice thickness = 1.0 mm without a gap, TR = 8.2 ms, TE = 3.2 ms, flip angle = 12°, matrix 256 × 256, and field of view = 240 mm), were acquired.

### Image analysis

The evaluation of white matter lesions was provided by T2 weighted and fluid attenuated inversion recovery images. The degree of white matter hyperintensity (WMH) load was rated visually on axial images by using the Fazekas scale (i.e., grade 1 [punctate], grade 2 [early confluent], or grade 3 [confluent]) in the periventricular and deep white matter regions (11). The sum of the periventricular and deep WMH scores, ranging from 0 to 6, was used for the analysis.

The following image processing steps were applied for analysis of DTI data on the cortical surface, as described in detail elsewhere (12–16). The native MRI data of all subjects were spatially normalized to the stereotaxic space and corrected for intensity non-uniformity artifacts (12). A hierarchical multi-scale non-linear fitting algorithm was then applied to normalize the skull-stripped MR images by a brain extraction tool and to provide *a priori* information, that is, tissue probability maps for subsequent tissue classification using the neural network classifier (12). Partial volume errors due to tissue-mixing at their interfaces were estimated and corrected using the trimmed minimum covariance determinant method (15). Hemispheric cortical surfaces were automatically extracted from each MR volume using constrained Laplacian-based automated segmentation with the proximities (CLASP) algorithm, which reconstructed the inner cortical surface by deforming a spherical mesh onto the white matter boundary and then expanded the deformable model to the gray matter boundary (14). All DTI data were preprocessed using FMRIB’s software Library program^1^. First, head motion artifacts and eddy current distortions in the DTI data were corrected by applying an affine transform to their first non-diffusion-weighted (b0) image. Then, skull stripping was performed by removing non-brain structures using the brain extraction tool. Thereafter, MD maps were estimated by fitting a diffusion tensor model to each voxel of the preprocessed DTI data. Volumetric MD maps were directly mapped to the corresponding intermediate cortical surface, halfway between the inner and outer CLASP surfaces as it represents a relatively unbiased representation of both sulcal and gyral regions, using the nearest-neighbor projection method (16). We employed an iterative surface registration algorithm to ensure an optimal correspondence at each vertex of the cortical surface model across individuals (13). Diffusion smoothing that generalized Gaussian kernel smoothing was used with a 30 mm full width at half maximum kernel to augment the signal-to-noise ratio and optimally detect changes in population.

The lateral ventricles were segmented using a fully automated method based on a graph cuts algorithm combined with atlas-based initialization and morphological postprocessing (Supplementary Material MRI Methods).

### Statistical analysis

R version 4.0.3^2^ was used for statistical analysis. The mean MD values for INPH-specific areas showing distinctive cortical MD changes of the INPH, AD, and control groups were compared by analysis of variance or Kruskal-Wallis tests, followed by Tukey’s *post hoc* analysis. Diagnostic accuracy of the mean MD in INPH-specific areas showing distinctive cortical MD changes for distinguishing INPH from AD was calculated using area under the curve, sensitivity, specificity, and cutoff levels obtained using receiver operating characteristic (ROC) curves. Statistical significance was set at *p* < 0.05.

## Results

We enrolled 42 patients with INPH, 51 patients with AD, and 23 healthy controls. Patients and controls are characterized in Table 1. There were no significant age differences between the three groups. There were no significant differences in Fazekas scores between the AD and control groups.

**Table 1:** Characterization of patients and controls at baseline.

Characteristics	Controls (*n* = 23)	INPH (*n* = 42)	AD (*n* = 51)
Age (year)	71.5 ± 4.2	72.9 ± 5.3	71.0 ± 8.1
Gender, male	8 (34.8)	26 (61.9)	14 (27.5)
Education (year)	11.9 ± 5.0	9.2 ± 4.7	7.7 ± 4.6
Duration of symptoms (year)		2.8 ± 2.5	2.3 ± 1.3
K-MMSE	27.2 ± 2.3	20.1 ± 5.6	18.6 ± 4.4
Fazekas score	2.7 ± 1.6	4.3 ± 1.3	2.9 ± 1.7

Values denote number (%) or mean ± SD.

INPH, idiopathic normal-pressure hydrocephalus; AD, Alzheimer’s disease; K-MMSE, Korean version of Mini-Mental State Examination.

**Figure 1:**
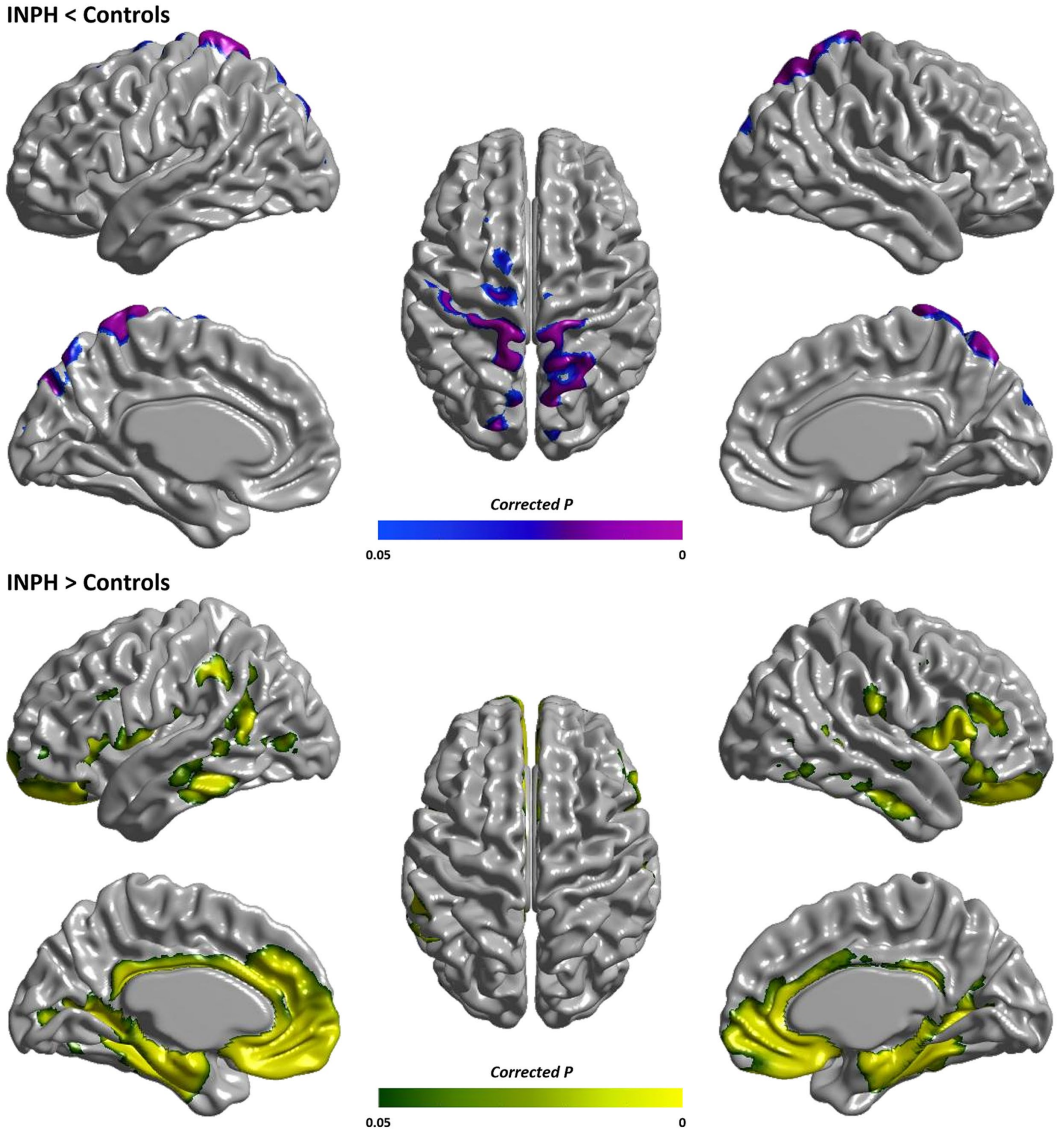
Group comparison of cortical MD between INPH patients and healthy controls. Surface maps represent cortical clusters where INPH patients showed either significantly reduced (blue-purple) or increased (green-yellow) MD compared to healthy controls (*p* < 0.05, corrected for multiple comparisons with false discovery rate). MD, mean diffusivity.

Relative to age- and sex-matched healthy controls, INPH patients demonstrated statistically significant lower MD in the high convexity of the frontal, parietal, and occipital cortical regions (FDR-corrected *p*-value, pFDR <0.05, Figure 1). We designate these clusters of lower MD as INPH MD LOW ROI. Furthermore, INPH patients demonstrated statistically significant higher MD mainly in the ventromedial frontal cortex, ventrolateral frontal cortex, supramarginal gyrus, and temporal cortical regions relative to controls (pFDR <0.05, Figure 1). We designate these clusters of higher MD as INPH MD HIGH ROI.

**Figure 2:**
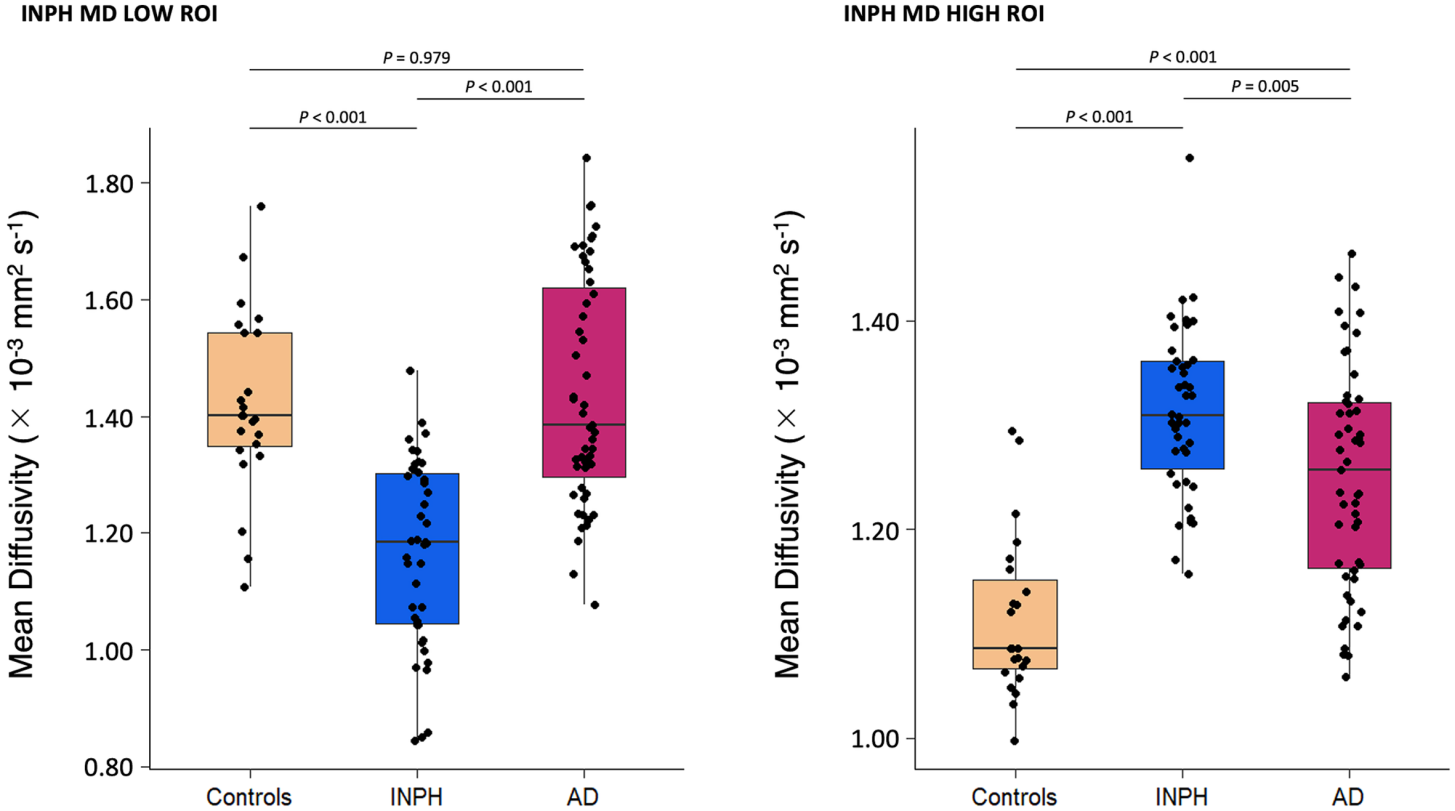
Mean MD values for the brain regions with significant clusters obtained on cortical surface-based DTI analyses in Figure 1. In INPH MD LOW ROI, INPH patients, when compared to AD and control groups, showed a statistically significant decrease in average MD values. In INPH MD HIGH ROI, INPH patients, when compared to AD and control groups, showed a statistically significant increase in average MD values. DTI, diffusion tensor imaging; MD, mean diffusivity; ROI, region of interest.

We calculated mean MD values for INPH MD LOW ROI and INPH MD HIGH ROI for each participant. For the INPH MD LOW ROI analysis, INPH patients demonstrated a statistically significant decrease in mean MD when compared to AD and control groups; however, there were no significant differences in mean MD values between the AD and control groups (Figure 2). For the INPH MD HIGH ROI analysis, INPH patients demonstrated a statistically significant increase in mean MD when compared to AD and control groups; further, AD patients showed a statistically significant increase in mean MD when compared to controls (Figure 2). We assessed the associations between the mean MD values of the INPH MD LOW and HIGH ROIs in the INPH group and both clinical parameters and normalized lateral ventricle volume (Supplementary Table 1). TUG scores were positively correlated with the mean MD values for the INPH MD HIGH ROI.

**Figure 3:**
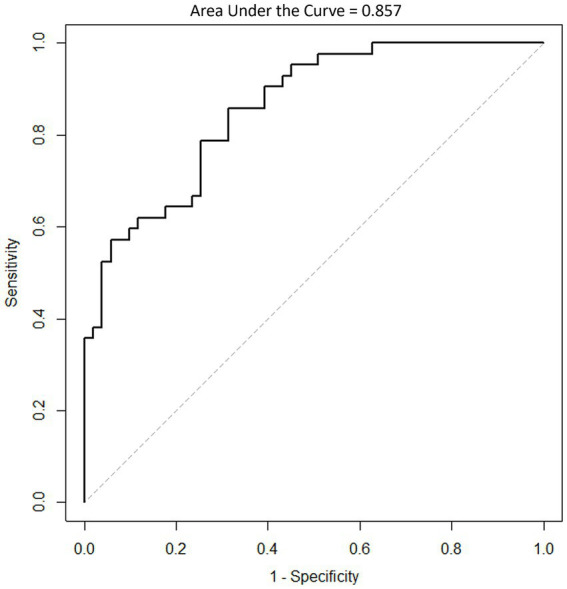
Receiver operating characteristic (ROC) curve in classifying INPH patients and AD patients using the mean MD value for INPH MD LOW ROI. MD, mean diffusivity; ROI, region of interest.

The mean MD value for INPH MD LOW ROI of the INPH group was 1.17 ± 0.16 × 10^−3^ mm^2^ s^−1^ and the mean MD value for INPH MD LOW ROI of the AD group was 1.44 ± 0.19 × 10^−3^ mm^2^ s^−1^ (mean ± standard deviation), a significant difference (*p* < 0.001). The ROC curve showed that a cutoff score of 1.32 × 10^−3^ mm^2^ s^−1^ on the mean MD value for INPH MD LOW ROI yielded the highest sensitivity and specificity with regard to differentiating patients with INPH and AD (Figure 3). Moreover, the area under the ROC curve was 0.857, indicating that this ratio had a good discriminant ability.

## Discussion

Compared with age- and sex-matched healthy controls, INPH patients showed statistically significant lower MD in the high convexity of the frontal, parietal, and occipital cortical regions. Importantly, in this INPH MD LOW ROI, INPH patients showed a statistically significant decrease in mean MD when compared to AD. Additionally, we observed significantly higher MD mainly in the ventromedial frontal cortex, ventrolateral frontal cortex, supramarginal gyrus, and temporal cortical regions in the INPH group relative to controls. And in this INPH MD HIGH ROI, INPH patients showed a statistically significant increase in mean MD when compared to AD. These results provide some evidence for a characteristic pattern of cortical MD changes in INPH patients.

As a hypothesis about the cause of decreased cortical MD in our INPH patients, we might speculate as follows. First, cortical MD reduction has been observed in patients with other neurodegenerative diseases. For example, a study of autosomal-dominant AD mutation carriers showed that cortical MD was reduced in presymptomatic mutation carriers compared to non-carriers in clusters including parietal, frontal, temporal, and occipital regions (17). As another example, in early preclinical AD, there was decreased MD in the left and right inferior and middle temporal gyrus, and in the right superior parietal areas (6). Second, in other neurodegenerative diseases, it has been suggested that cortical MD reduction could be a consequence of neuroinflammation and reactive gliosis that often follow brain pathologies. One recent study found a negative association between astrocytosis, as measured by ^11^C-deuterium-L-deprenyl PET binding, and cortical MD in autosomal-dominant AD mutation carriers, which may suggest that the inflammatory process can produce changes in cell phenotype including increased cell number (glial recruitment and activation) that can explain the decrease in cortical MD (17). Reactive gliosis is well known as the cellular manifestation of neuroinflammation (18). Any insult to the central nervous system tissue, including neurodegenerative diseases, also triggers reactive gliosis (19). Third, neuroinflammation and reactive gliosis are generally understood to be associated with hydrocephalus (20). Hydrocephalus-related ventricular enlargement may lead to compression of the surrounding brain parenchyma, thereby triggering reactive gliosis characterized by the proliferation of microglia and astrocytes (20). Notably, patients with INPH demonstrate marked surface expansion primarily in the superior portion of the bilateral lateral ventricles, which are encased by the medial frontal lobe and the high convexity of the frontal and parietal regions, and the medial portions of the frontal horns (21). While patients with INPH demonstrate marked ventricular dilatation, compression of the CSF spaces over the high convexity and midline areas has been hypothesized to be a key imaging feature of the disease, i.e., disproportionately enlarged subarachnoid space hydrocephalus (DESH) (22). Consequently, these regions might deserve inclusion in the key areas for INPH disease progression. To our knowledge, no study has reported cortical MD measurement in INPH patients. In our study, we cautiously hypothesize that cortical MD reduction in INPH may result from neuroinflammation and reactive gliosis. Further, regarding the characteristic regional pattern of decreased cortical MD in our INPH patients, we note that some studies report hyperperfusion in high convexity areas in INPH (4, 23), and hyperperfusion may also be a phenomenon related to neuroinflammation (4, 24–26).

As an explanation for characteristic patterns of increased cortical MD in our INPH patients, we might speculate as follows. Cortical MD increase is known to be a biomarker of neurodegenerative changes in AD and other neurodegenerative diseases (17, 27–31). It was reported that, upon neuronal loss, water can diffuse more freely within the cerebral cortex, thereby increasing intracortical MD values (29, 30). And cortical MD has been found to increase across multiple regions in AD, behavioral variants of frontotemporal dementia, and primary progressive aphasia patients (27–29). Regarding the characteristic regional pattern of increased cortical MD in our INPH patients, we note that some studies report hypoperfusion in frontal and temporal areas in INPH (4, 23), and hypoperfusion can be a phenomenon related to neuronal degeneration (4, 32, 33). In our study, cortical MD increase in INPH might result from neurodegeneration.

The mean MD value for INPH MD LOW ROI was markedly different between INPH and AD groups in our study. Diagnosing INPH can be difficult as symptoms of INPH are common in the elderly and often overlap with symptoms of other neurodegenerative disorders such as AD (34). For example, gait disturbance is also common in patients with AD, which further makes the differential diagnosis between AD and INPH difficult (35). Moreover, non-obstructive enlargement of the cerebral ventricles in INPH can be difficult to distinguish from age- and AD-related ex vacuo ventricular enlargement by conventional CT and MR imaging techniques (34). AD is generally considered a cortical disease (36). For example, early neurodegeneration involving cortical gray matter is a recognized feature of AD (31). And it has been reported that cortical diffusion measures are useful in differentiating AD from other types of dementia (37, 38). However, in INPH patients, white matter changes are common, and cortical changes are often overlooked (2, 39). Nevertheless, ventricular surface expansion in INPH is also associated with cortical structural changes (21). Therefore, it might be possible to distinguish INPH from AD using the mean MD value for INPH MD LOW ROI. However, further studies with larger study populations and various statistical tools would be needed to establish this value for cortical structural changes as a neuroimaging biomarker to distinguish INPH from AD. And, as mentioned above, reductions in cortical MD have been reported in AD, particularly during its early stages (6, 17). The AD patients included in our study were diagnosed with clinically probable AD dementia, which may represent a more advanced stage where such changes are less prominent or more variable. Further studies involving a larger cohort and applying AD biomarker-based staging are needed to clarify these findings.

The INPH patients were chosen consecutively from a prospectively enrolled INPH registry at our hospital. One limitation of this study is that INPH patients with a negative CSFTT response were not included. This was done to enhance diagnostic certainty of INPH. In accordance with the Japanese guideline, clinical improvement after the CSFTT increases diagnostic certainty from possible to probable (9). Moreover, INPH patients with a negative CSFTT response are more likely to have other cerebral comorbidities (40). An additional limitation was that AD-specific biomarkers were not determined in this study. Although AD-type dementia diagnosis was established according to the NIA-AA guidelines, the diagnosis was not confirmed with amyloid biomarker evidence. AD biomarkers were grouped as pathologic tau, β-Amyloid (Aβ) deposition, and neurodegeneration (41). Aβ and pathologic tau biomarkers show specific neuropathologic changes that determine AD (41). Staging can be done by combining information from the three biomarker groups. In this case, a more advanced pathologic stage is associated with more abnormal biomarker groups (41). Further, AD pathology could not be determined in the INPH patients. Problems with CSF can disrupt elimination of toxic metabolites and can result in accumulation of amyloid peptides (42). Moreover, CSF stasis in INPH patients may also show patterns of brain atrophy consistent with AD (21). That said, we believe investigating cortical MD utilizing surface-based analysis in a large study of INPH patients is warranted. A final limitation was that additional biomarkers of brain injury, including fluorodeoxyglucose positron emission tomography, or biomarkers associated with neuroinflammation were not measured in our study. As a result, neuronal degeneration and reactive gliosis could not be measured in our participants. However, to the best of our knowledge, there has been no study that analyzes differences in cortical MD between INPH patients and healthy controls that utilizes whole-brain vertex-by-vertex analysis.

A distinctive pattern of cortical MD changes was found in INPH patients, and cortical regions of low MD distinguished INPH from AD with good diagnostic sensitivity and specificity. Our findings suggest microstructural changes in cortical integrity can help differentiate INPH and AD in elderly patients.

## Data Availability

The raw data supporting the conclusions of this article will be made available by the authors, without undue reservation.

## Footnotes

^1^ http://fsl.fmrib.ox.ac.uk/fsl/fslwiki/FSL

^2^ https://www.r-project.org
