# Supplementary material for: Distinct cerebral cortical microstructural changes in idiopathic normal-pressure hydrocephalus
Source: Front Neurol. 2025 Aug 13;16:1618788. doi: 10.3389/fneur.2025.1618788 (PMC12381526; doi:10.3389/fneur.2025.1618788)
Supplement: Supplementary file 1 [file Supplementary_file_1.docx]

Supplementary Material

# Supplementary Table 1. Correlations of clinical measures and normalized lateral ventricle volume with mean MD values of INPH MD LOW and HIGH ROIs in INPH patients.

|  | Correlation coefficients and P values | |
| --- | --- | --- |
|  | Mean MD value of INPH MD LOW ROI | Mean MD value of INPH MD HIGH ROI |
| Timed Up and Go test | 0.143 (0.365) | 0.386 (0.012)* |
| K-MMSE | -0.155 (0.328) | -0.280 (0.073) |
| INPH Grading Scale, Urinary function | -0.033 (0.838) | 0.149 (0.346) |
| Normalized lateral ventricle volume | -0.126 (0.428) | 0.050 (0.752) |

*Statistically significant relationships.

Normalized lateral ventricle volume = (lateral ventricle volume)/(intracranial volume)

MD = mean diffusivity; INPH = idiopathic normal-pressure hydrocephalus; ROI = region of interest; K-MMSE = Korean version of Mini-Mental State Examination.

**Supplementary MRI Methods**

We adopted a graph cuts algorithm combined with atlas-based segmentation to delineate the lateral ventricles, utilizing a priori information for the graph cut process (1). A predefined lateral ventricle label from the ICBM 152 symmetric template was nonlinearly registered to each individual scan, providing foreground seeds for the graph cuts algorithm (1). These seeds enabled the identification of a global minimum of the energy function via the minimum cut/maximum flow algorithm. The initial seed definition was further refined using partial volume estimation at each voxel. Finally, the segmentation output was post-processed using morphological opening to reduce over- and under-segmentation artifacts (2).

**Supplement References**

1. Park S, Yoon U. Automated Segmentation of the Lateral Ventricle Based on Graph Cuts Algorithm and Morphological Operations. *J Biomed Eng Res.* (2017) 38(2):82-8. doi: 10.9718/JBER.2017.38.2.82

2. Kwak K, Yoon U, Lee DK, Kim GH, Seo SW, Na DL, et al. Fully-Automated Approach to Hippocampus Segmentation Using a Graph-Cuts Algorithm Combined with Atlas-Based Segmentation and Morphological Opening. *Magn Reson Imaging.* (2013) 31(7):1190-6. doi: 10.1016/j.mri.2013.04.008
